# High pyocyanin production and non-motility of *Pseudomonas aeruginosa* isolates are correlated with septic shock or death in bacteremic patients

**DOI:** 10.1371/journal.pone.0253259

**Published:** 2021-06-11

**Authors:** Asmita Gupte, Jeevan Jyot, Malleswari Ravi, Reuben Ramphal

**Affiliations:** 1 Division of Infectious Diseases and Global Medicine, Department of Medicine, University of Florida, Gainesville, Florida, United States of America; 2 Department of Pharmaceutical Outcomes & Policy, College of Pharmacy, University of Florida, Gainesville, Florida, United States of America; COMSATS University Islamabad - Abbottabad Campus, PAKISTAN

## Abstract

Studies of the outcome of *Pseudomonas aeruginosa* bacteremia (*Pab)* have focused mainly on antibiotic appropriateness. However, *P*. *aeruginosa* possesses many virulence factors whose roles in outcomes have not been examined in humans, except for the type III secretion system (T3SS) toxins. The purpose of this study was to examine the role of virulence factors other than the T3SS toxins. Bacterial isolates were collected from 75 patients who suffered from *Pa* blood stream infections. Host factors such as neutropenia, immunosuppression, comorbidities, time to effective antibiotics, source of bacteremia, and presence of multidrug resistant (MDR) isolate were studied. The isolates were analyzed for the presence of toxin genes, proteolytic activity, swimming and twitching motility, and pyocyanin production. The data were analyzed to ascertain which virulence factors correlated with poor outcomes defined as septic shock or death (SS) within 7 days. Septic shock or death occurred in 25/75 patients. Univariate analysis identified age as a host factor that exerted a significant effect on these outcomes. Ineffective antibiotics administered during the first 24 hours of treatment or MDR *P*. *aeruginosa* did not influence the frequency of SS, nor did the presence of *lasB*, *exoA*, *exoS exoU*, *plcH* genes and proteolytic activity. However, 6/8 patients infected with non-motile isolates, developed SS, p = 0.014 and 5/6 isolates that produced large amounts of pyocyanin (>18ug/ml), were associated with SS, p = 0.014. Multivariate analysis indicated that the odds ratio (OR) for development of SS with a non-motile isolate was 6.8, with a 95% confidence interval (CI) (1.37, 51.5), p = 0.030 and with high pyocyanin producing isolates, an OR of 16.9, 95% CI = (2.27, 360), p = .017. This study evaluating the role of microbial factors that significantly effect outcomes following *Pa* bloodstream infection suggests that *P*. *aeruginosa* strains showing high pyocyanin production and the lack of motility independently increase the risk of SS.

## Introduction

*P*. *aeruginosa* is reported to cause the highest mortality rate among common gram negative bloodstream infections (BSI) [1, 2]. The appropriateness of antibiotic therapy and host factors have been implicated in determining its outcome [3–5], but there appear to be other elements involved, since patients die from *Pab* even when treated appropriately [6] and patients treated inappropriately, survive. Among host factors, a pulmonary focus carries the highest mortality rate [3]. Neutropenia has also been strongly associated with deaths [7, 8]. Other host factors implicated generally reflect multiorgan dysfunction that is likely to contribute to mortality. Thus far, persistent neutropenia is the best defined host factor that affects the outcome of *Pab* [7, 8].

The role that the different virulence factors of *P*. *aeruginosa* play in poor outcomes in humans has received comparatively little attention although there are a plethora of animal studies aiming to define the virulence factors that play roles in death due to *P*. *aeruginosa* [9–11]. In animals, the products of the type III and type II secretion (T3SS and T2SS respectively) systems of *P*. *aeruginosa* have been implicated in death due to lung infections [12–15]. In humans, there is also evidence that the T3SS associated toxins may be involved in death due to lung infections [16, 17]. However, there is a paucity of data concerning role of bacterial factors involved in poor outcomes in BSI. One study examined 30-day mortality following *Pab* and concluded that the presence of a functioning T3SS was highly associated with death [18]. A second study reported that the presence of the *exoU* gene that encodes a phospholipase toxin, secreted by the T3SS of *P*. *aeruginosa* is significantly associated with early deaths in blood stream infections [19], but not with overall mortality. However, not all early deaths or the occurrence of septic shock could be explained by the presence of this genotype or failure of antibiotic therapy, implying that other virulence factors may be involved [20, 21]. The main purpose of this study was therefore to broadly examine the virulence factors of isolates from patients with poor outcomes from *Pab*. We define poor outcomes as the development of septic shock or death within a 7-day period from the day a positive blood culture was obtained. The rationale for using this time frame being that septic shock is the antecedent event to death, but many patients survive septic shock with the advanced supportive care measures currently used, despite having what can be regarded as a poor outcome. Additionally, among patients who die beyond the 7-day period, death may be due to a comorbid condition. In this study we evaluated host factors such as neutropenia, immunosuppression, comorbidities, time to effective antibiotics, duration and source of bacteremia, as well as presence of MDR *P*. *aeruginosa* isolates. Further, we examined the presence or absence of virulence factors genes such as: *lasB*, which encodes the major secreted elastase of *Pa; exoA*, which encodes exotoxinA*; exoS* and *exoU* which encode the major toxins secreted by T3SS; and *plcH*, which encodes the hemolytic phospholipase. Phenotypic analysis of these virulence factors was not performed. It was not our purpose to reexamine antibiotic therapy for *Pab* in depth, since this has been exhaustively examined [22].

## Materials and methods

### Study design and subjects

The first blood isolates from patients with a laboratory diagnosis of *Pab* were retrieved from the microbiology laboratory within a week of bacteremia and frozen at -70°C in 25% sterile glycerol. Following discharge or death of the patients, their medical records were examined and demographic data, data pertaining to underlying diseases, treatment and outcomes were extracted from the charts. The study was approved by the Institutional Review Board (IRB) of University of Florida (UF) and Shands Hospital, which at that time was a 625-bed tertiary care primary teaching hospital for the University of Florida, College of Medicine, with the full complement of Medical and Surgical Specialties. Informed consent was waived by the UF IRB. No patient suffered more than one episode of *Pab*. Patient data and bacterial isolates were collected between October 2007 and July 2009. Patients <18 years of age, pregnant females and patients with polymicrobial bacteremia were excluded.

### Definitions

**Bacteremia** was defined as ≥1 blood culture growing *P*. *aeruginosa*. The duration of bacteremia was scored as the number of days the blood cultures remained positive.

**Septic shock** was defined as hypotension with systolic blood pressure of <90 mm Hg or mean arterial pressure <65 mm Hg, that was unresponsive to fluid administration alone, in the setting of a positive blood culture for *P*. *aeruginosa*.

**Effective antibiotic therapy** was defined as the administration of at least one antibiotic to which the infecting bacterium was susceptible, within a 24-hour period of the positive blood culture. No patient received an aminoglycoside alone as primary therapy.

**Multidrug resistance** was defined as resistance to three single anti-Pseudomonal drugs within three different classes of antibiotics, including aminoglycosides, the anti-Pseudomonal cephalosporins, carbapenems, and antipseudomonal penicillin/beta-lactamase inhibitors.

**Sources of the infection** were based on clinical assessment or site from which the organism was isolated. Sources were classified as abdomen, burns/wound, line/endovascular, pneumonia/lung, urine, and unknown.

**Comorbidities** were noted as the type of underlying diseases, e.g. cancer, burns, kidney disease, diabetes mellitus (DM), gastrointestinal disease (GI disease), solid organ transplantation (SOT), cardiovascular disease and other.

**Neutropenia** was defined as <500 neutrophils/*u*l of blood at the time the blood cultures were obtained.

**Immunosuppression** was defined as compromised immune system due to cancer therapy, SOT immunosuppression, biologics, or chronic steroid therapy (prednisone dose equivalent of 5 mg or more for more than 3 months).

### Detection of genes encoding the major *Pseudomonas* toxins

Polymerase chain reaction (PCR) assays for detection of *exoS*, *exoU*, *exoA*, *lasB*, *plcH* genes were performed with primers and protocol described by Feltman *et al* [23] with modifications. *P*. *aeruginosa* isolates were grown as individual colonies on LB agar plates. A single colony was then inoculated in LB medium and grown overnight at 37°C. Chromosomal DNA was extracted using Promega kits (catalog no. A1120). Primers for *exoA*, *lasB*, *plcH*, *exoS*, *and exoU* genes were designed based on known gene sequence and synthesized by Geno-Mechanix, Gainesville, Florida (S1 Table). PCR reactions were performed with AmpliTaq DNA polymerase in a DNA thermal cycler (Thermo Fisher), under the following conditions: denaturation for 10 minutes at 95°C, followed by 35 cycles of 90°C for 60 seconds and 70°C for 4 minutes, and a final extension step of 10 minutes at 72°C. Chromosomal DNA from *P*. *aeruginosa* laboratory strains PAK (wild-type laboratory strain/motile which carries *exoS*, and PA14 (carries *exoU*) were used as positive controls to detect the presence of these two toxin genes, the two genes being almost always being mutually exclusive in the same strain [23]. For isolate 87, PCRs did not work despite repeated efforts. PCR products were electrophoresed through agarose gels (0·8%, w/v) containing ethidium bromide (0·5 μg/ml) and visualized using UV light (S1 and S2 Figs).

### Swimming and twitching motility

*P*. *aeruginosa* clinical isolates and controls were grown overnight on Luria agar plates at 37°C. A single colony was used for further evaluation and experiments were performed in triplicates. The swimming motility (referred to simply as motility in further sections) of different *P*. *aeruginosa* strains was assessed qualitatively by examining the circular swarm from the growing motile bacterial cells on 0.325% agar plates at 37°C after point inoculation breaking the surface of agar and scored as motile or non-motile [24]. Strain PAK and its non-motile mutant [25] served as positive and negative controls.

Twitching motility, indicative of the presence of functioning Pseudomonas pili was ascertained using point inoculation of single colony, in triplicates, with a sterile toothpick at the bottom of 1% Luria agar plates [Difco LB broth (10 g/liter tryptone/5 g/liter yeast extract/10 g/liter NaCl) solidified with 1% (wt/vol) Difco granulated agar]. After incubation at 37°C for 24 h, the zone of motility at the agar/Petri dish interface was measured as present or absent [26].

### Proteolytic activity

Each clinical isolate and the control PAK strain were grown on Luria agar plates with overnight incubation at 37°C. The proteolytic activity of each *P*. *aeruginosa* strain was determined on skim milk agar by inoculating the surface with a sterile toothpick performed. The assay was performed in triplicate for each strain. The diameter of the zone of proteolysis as indicated by clearing of the milk agar was noted after 24 h of incubation at 37°C [27].

### Pyocyanin production

Pyocyanin production was assayed on the basis of the absorbance of pyocyanin at 520 nm in an acidic solution. Five ml of supernatant from a stationary-phase culture (16 h) in Luria broth was mixed with 3 ml of chloroform after adjustment for the background of optical density (OD). Pyocyanin from the chloroform phase was then reextracted into 1 ml of 0.2 N HCl giving a pink to deep red solution. Concentrations, expressed as micrograms of pyocyanin produced per milliliter of culture supernatant, were determined by multiplying the OD at 520 nm by 17.072 [28]. A consistently high pyocyanin producing clinical isolate, isolate 4- from our study and an isolate that did not produce pyocyanin, isolate 6, were used as positive and negative controls respectively for all pyocyanin measurements. Each blood stream isolate was evaluated in triplicate.

### Western blot analysis for flagellin and electron microscopic studies for flagella

The presence of flagellin in whole cell lysates was assessed using Western Blot Analysis. Bacterial cultures were grown in Luria broth overnight. The cells were centrifuged and the pellet was lysed in 1× SDS buffer (2% SDS, 1% β‐mercaptoethanol, 50mM Tris HCl, pH = 7.5) in one‐tenth of the culture volume and boiled. The lysed extracts were electrophoresed on a 10% SDS‐polyacrylamide gel. The proteins were transferred by electro transfer to 0.45μm size PVDF membranes. The blots were developed using rabbit anti-flagellin polyclonal antibody as primary antibody [25]. Alkaline phosphatase‐conjugated anti‐rabbit IgG was used as the secondary antibody (Bio-Rad 170–6518). The blots were developed using NBT and BCIP color substrates using Bio-Rad Kit (catalog # 170–6532). PAK-D (PAK *fli*D mutant that overexpresses flagellin) [29] was used as positive control and PAKΔ*fliC* mutant [30] that does not produce flagellin was used as negative control (S3 Fig). All non-motile strains were examined by electron microscopy after negative staining, to evaluate for presence of flagellum. PAK and its non-motile mutant were used as positive and negative controls respectively. Static cultures were grown overnight at 37°C. A drop of the culture was allowed to adhere to a carbon-coated grid for 10s and drained off; the grid was then rinsed in a drop of saline, and adherent cells were negatively stained with a 2% aqueous solution of phosphotungstic acid for 10s. Samples were examined with a Hitachi H-7000 transmission electron microscope [31]. The EM was performed by the EM laboratory at the University of Florida ICBR. Strains that did not demonstrate flagella were re-evaluated to verify the absence of flagella with a wild type control strain PAK used each time to ensure validity of the manipulation.

### Statistical analysis

R statistical software package (V. 3.0.2, Vienna, Austria) was used to calculate descriptive statistics and perform univariate tests. P-values are the result of Fisher’s exact tests, t-tests or Mann-Whitney tests, as appropriate. Multivariate analysis was performed using logistic regression to assess the effects of potential predictors of SS when controlling for age, source of the bacteremia and total number of comorbidities.

## Results

During a 20-month period, 101 patients had positive blood cultures for *P*. *aeruginosa*. After exclusions, data from 75 patients and their respective bacterial isolates were included in the analysis. Based on chart reviews and application of the definitions, 25/75 patients were classified as having poor outcomes (septic shock or death within a 7-day period, referred to as the SS group in later sections) and 50/75 in a no shock group (NSS). The mortality in the SS group was 64% versus 6% in the NSS group. The overall mortality for all patients at 30 days was 25%.

### Host factors

#### Univariate analysis

(Table 1) The mean age of patients in the SS group was 62.5 years and NSS group 54.1 years (p = 0.014). There were no statistically significant differences between the 2 groups in host factors such as WBC count, immunosuppression or neutropenia or both. Comorbidities such as cancer, burns, kidney disease, diabetes mellitus, gastrointestinal diseases, SOT, cardiovascular disease did not appear to influence the occurrence of SS. In contrast, the site of infection showed a statistically significant influence on the development of SS. Nine of the 16 (56.2%) patients who had pneumonia as the source of *Pab* developed SS whereas 16 out of 59 (27.1%) patients with all other sources, developed SS (p = 0.038).

**Table 1:** Univariate analysis of host factors as predictors of septic shock and/or death in 7 days.

	SS	NSS	p-value
	n = 25 (%)	n = 50 (%)	
**Age**			
**Mean (SD)**	62.5 (16.7)	54.1 (16.7)	.046
**Median [IQR] (range)**	57 [52, 77] (35, 88)	57.5 [43.5, 66] (19, 85)	
**Female, n (%)**	13 (52.0)	18 (36)	.281
**WBC**			
**Mean (SD)**	10.7 (6.8)	9.2 (7.9)	.379
**Median [IQR] (range)**	10.7 [7.3, 16.1] (0.10, 22.5)	7.7 [3.2, 12.9] (0.10, 45.8)	
**Immunocompromise, Neutropenia or both**	12/25 (48)	22/50 (44)	0.808
**Source of bacteremia: Pneumonia**	9/25 (36)	7/50 (14)	0.038
**Cancer**	9/25 (36)	9/50 (18)	0.096
**Burns**	2/25 (8)	5/50 (10)	1
**DM**	2/25 (8)	9/50 (18)	.318
**SOT**	4/25 (16)	10/50 (20)	.763

SD, Standard deviation; IQR, Interquartile range; WBC, White blood cell count (x10^3^/mm^3^); SOT, Solid organ transplantation.

### Treatment

The effect of treatment factors was analyzed in a univariate analysis (Table 2). There were no significant differences in the time of receipt of effective antibiotic(s), the percentage of patients receiving treatment with ineffective antibiotics within the first 24 hours, the occurrence of MDR *P*. *aeruginosa* or duration of bacteremia between the SS group and the NSS group.

**Table 2:** Univariate analysis of effect of antibiotic therapy on the occurrence of septic shock.

	SS	NSS	p-value
	n = 25 (%)	n = 50 (%)	
**Effective Antibiotics within 24 h.**	20/25 (80)	37/50 (74)	0.775
**Time to Effective antibiotics in h.**	19.3 (20.9);	15.6 (20.2);	.355
**Mean (SD);**	13.5 [1.5, 24.8]	5.5 [0.4, 20.2]	
**Median [IQR] (range)**	(0, 72)	(0, 72)	
**MDR strains**	6/25 (24)	8/50 (16)	0.531

### Bacterial factors

The genes *lasB*, encoding elastase, *exoA*, encoding exotoxin A and *plcH*, encoding the hemolytic phospholipase of *P*. *aeruginosa*, were present in 100% of both SS isolates and NSS isolates thus their effect on outcomes could not be ascertained. Other bacterial factors such as twitching motility, total proteolytic activity which represents the combined actions of at least 3 known proteases [32], were not statistically significant determinants of SS. Since all strains from patients with or without SS carried the *exoS* or *exoU* gene, there was no control group to examine whether the occurrence of *exoS* or *exoU* was associated with a high rate of SS. Thus, we examined whether one or the other gene by itself was associated with SS. There was no association of these 2 individual genotypes with the occurrence of SS (Table 3).

**Table 3:** Univariate analysis of bacterial factors as predictors of septic shock and/or death (%) within 7 days.

	Bacterial factors present (% SS)	Bacterial factors absent (% SS)	p-value
**Swimming Motility**	19/67 (28)	6/8 (75)	.014
**Twitching Motility**	20/57 (35.1)	5/18 (27.7)	.775
**Proteolytic zone**	20/63 (31.7)	5/12 (41.6)	.610
exoS**	19/52 (36.5)	6/22 (27.2)	.592
exoU**	7/25 (28)	18/49 (36.7)	.604
**Pyocyanin >18 μg/ml**	5/6 (83.3)	20/69 (28.9)	.014

**Three isolates carried both *exoS* and *exoU* genes and for one isolate the PCR was unsuccessful hence the total number of strains carrying either of these genes appears to be 77 even if the total number of patients in the study was 75.

### Bacterial motility

**Fig 1:**
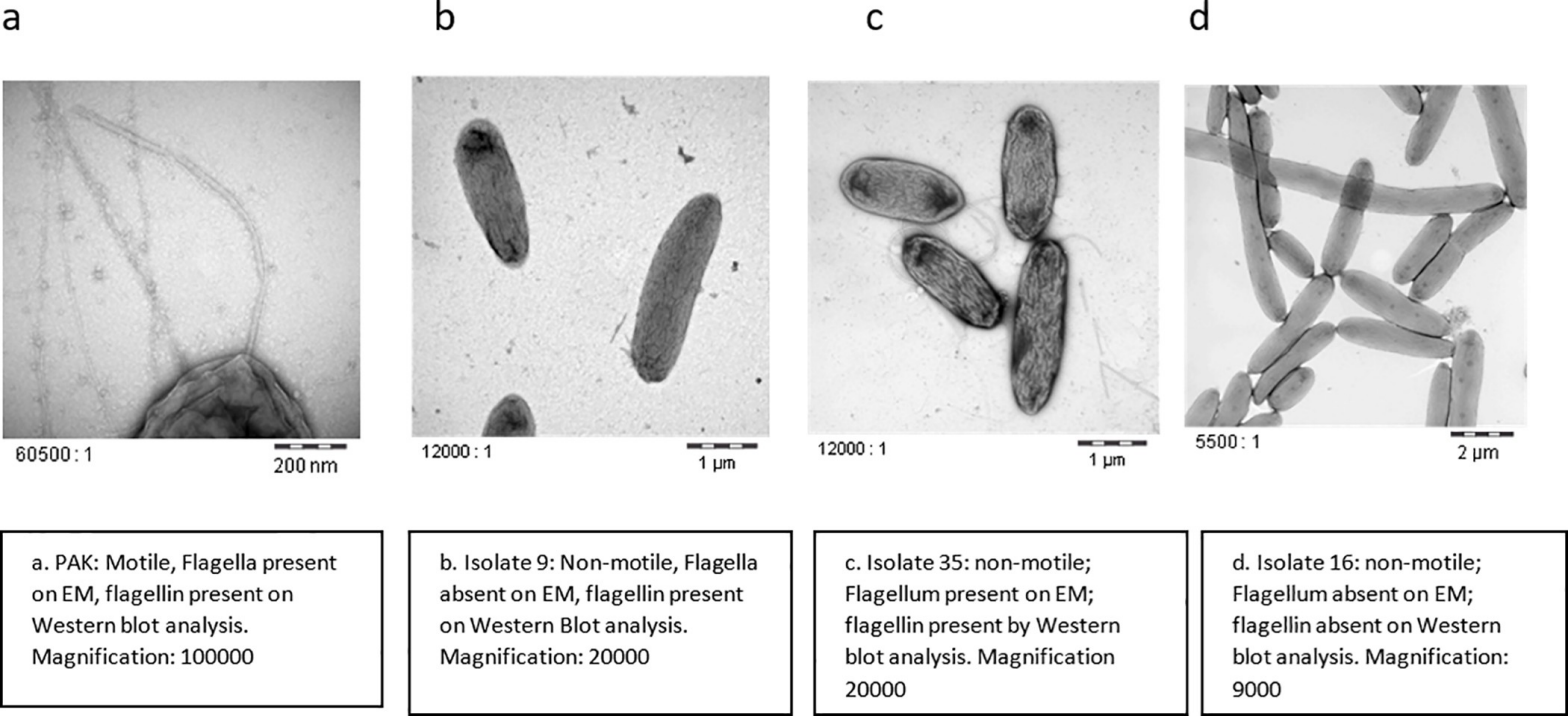
Electron microscopy images showing presence or absence of flagellum in select non-motile isolates and the control PAK strain.

*P*. *aeruginosa* displays a single polar flagellum which is essential for virulence in animal burn wounds [24, 33]. Surprisingly, 8/75 patients had BSI with non-motile *P*. *aeruginosa* isolates, 6 of whom developed SS (75%) p = 0.014 (Table 3). All 8 non-motile isolates were examined by electron microscopy and all, but one, lacked a flagellum (Table 4 and Fig 1). In the SS group (6 non-motile isolates), 2 isolates synthesized flagellin protein by Western blotting and one of these had a nonfunctional flagellum as noted on electron microscopy, presumably due to a mutation(s) in a motor or chemotaxis gene.

**Table 4:** Characteristics of non-motile strains with respect to development of SS, presence of flagellum by EM and presence of flagellin by Western blot analysis.

Isolate number	SS	Presence of Flagellum by EM	Presence of flagellin by Western Blot
**9**	Yes	No	Yes
**16**	Yes	No	No
**26**	No	No	No
35*	Yes	Yes	Yes
**40**	No	No	No
**62**	Yes	No	No
**77**	Yes	No	No
**79**	Yes	No	No

*The strain was confirmed to be non-motile on motility agar with repeated examination.

### Pyocyanin production

**Fig 2:**
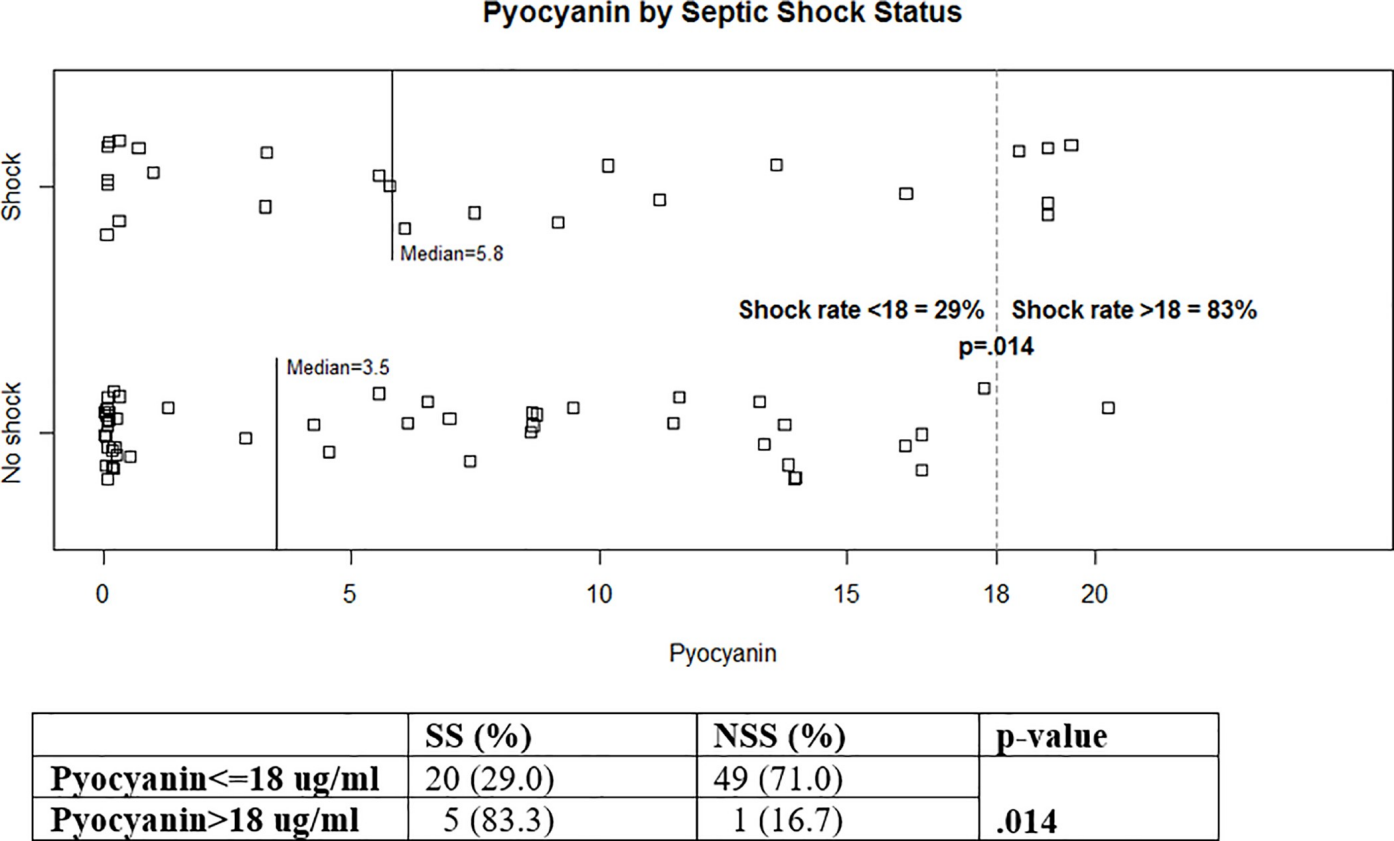
The Strip chart demonstrating pyocyanin levels (ug/ml) by septic shock status for all 75 subjects. The rate of SS was 83% among 6 subjects who had a pyocyanin level > 18 ug/ml. SS, septic shock or death within a 7-day period following *P*. *aeruginosa* bacteremia; NSS, no septic shock or death within 7-day period following *P*. *aeruginosa* bacteremia.

On visual examination of the strains on Luria agar, we noted that several patients were infected with strains that produced large amounts of pyocyanin. Most of these were isolated from patients who developed SS, however the median values for pyocyanin production of the all isolates from both groups of patients were not significantly different, (5.8 *u*g/ml for SS isolates versus 3.5*u*g/ml for NSS isolates (p = 0.189). Since pyocyanin production was a continuous variable, a Strip plot of pyocyanin production versus shock status was constructed (Fig 2). This plot showed a clear break in the data, with 5/6 (83%) of patient isolates that produced >18 ug/ml of pyocyanin causing SS, versus 20/69 (29%) of isolates that produced <18ug/ml (p = 0.014) (Table 3 and Fig 2).

### Motility and pyocyanin production

**Fig 3:**
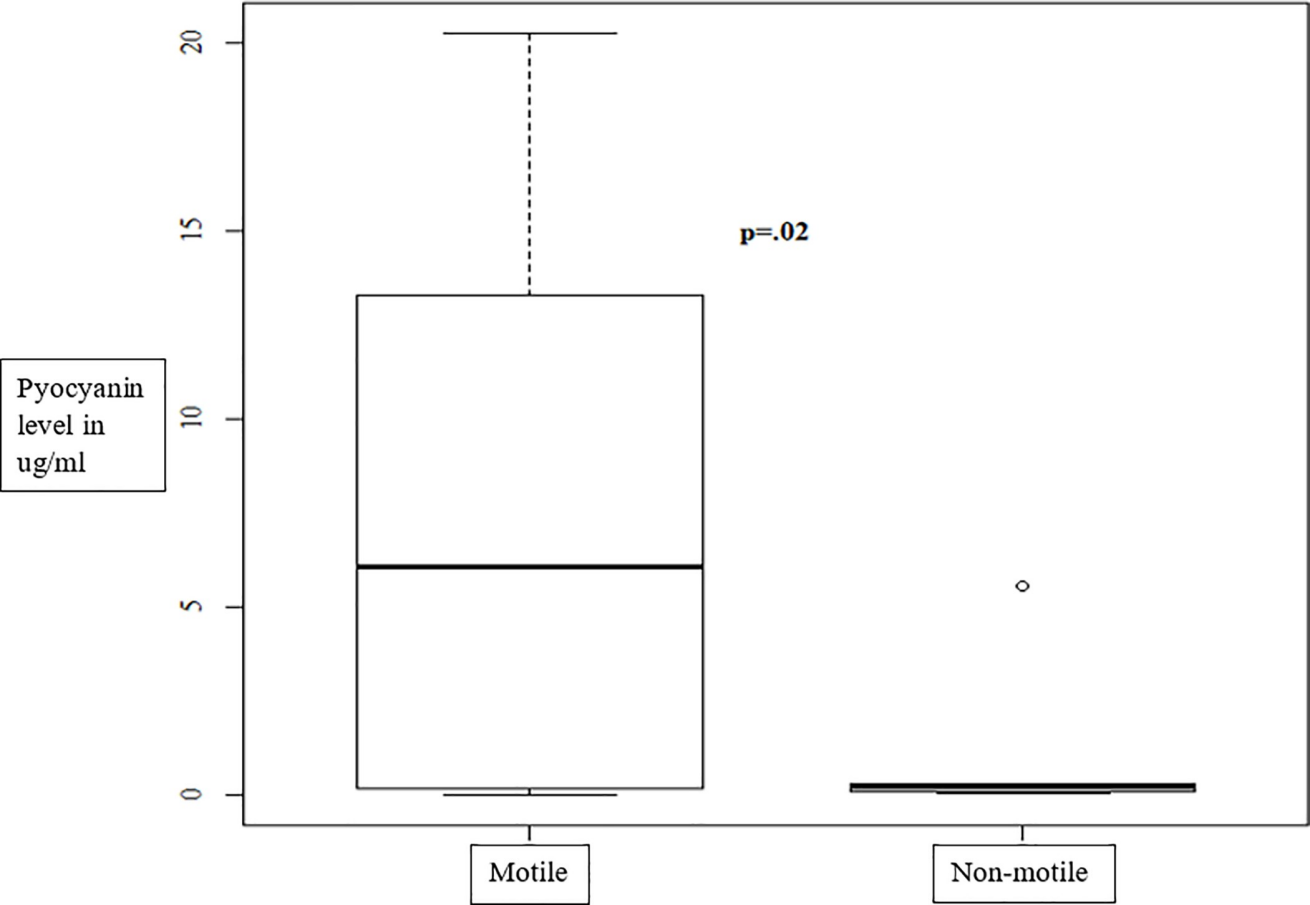
High pyocyanin production and non-motility are independent of each other in *P*. *aeruginosa* isolates associated with SS in bacteremia patients.

Since non-motility and high pyocyanin production were associated with SS, the concordance, if any, between these two factors was examined and is shown graphically in Fig 3. There was a statistically significant correlation between non-motility and low pyocyanin production (p = 0.02). All non-motile isolates produced less than 7 ug/ml of pyocyanin, most producing <2ug/ml. Thus, these microbial attributes were independent risk factors for the development of SS.

### Multivariate analysis

In the univariate analysis, 4 factors emerged as being significant; age, pneumonia, non-motility and high pyocyanin production. A multivariate analysis, controlling for age and number of comorbidities was therefore performed (Table 5). The small sample size precluded a multivariate analysis controlling for individual comorbidities. Age remained a risk for SS, P = .048. Pneumonia was marginally predictive of SS, OR = 3.2, 95% CI = [.988, 10.8], p = .054. The analysis also indicated that the OR for development of SS with a non-motile isolate was 6.8, 95% CI = (1.37, 51.5), p = 0.030 and that for pyocyanin values >18*ug*/ml, it was 16.9, 95% CI = [2.27, 360], P = .017 (Table 5). Due to the small sample size, we could not control for all factors in a multivariate analysis and hence we performed two more analysis controlling for age and pneumonia and separately looking at pyocyanin production and non-motility due to the inverse correlation between the two. Age, pneumonia and pyocyanin were examined in the first model. Age and high pyocyanin remained as risk factors. Each year of age increased the odds of death by 4%, age: OR = 1.04, CI = [1.00, 1.07], p = .047, pneumonia: OR = 3.0, CI = [.868, 10.7], p = .082, pyocyanin>18: OR = 16.0, CI = [2.05, 351], p = .021. Thus, pneumonia was marginally predictive of SS (p = 0.082). The second model, included age, pneumonia and non-motility; age: OR = 1.03, CI = [.995, 1.06], p = .117, pneumonia: OR = 3.0, CI = [.873, 10.5], p = .080, non-motility: OR = 6.2, CI = [1.19, 47.1], p = .041. Every year of age increased the risk of SS by 3% and once more pneumonia was marginally significant. High pyocyanin production and non-motility each remained strong independent risk factors for the development of SS during *Pab* and were the only virulence determinants among those we examined that carried an increased risk of SS or early death.

**Table 5:** Multivariate analysis using logistic regression controlling for age and number of comorbidities.

Host/Bacterial Factors	Estimated effect, p-value
**Age**	OR multiplies by 1.03 for each additional year, 95% CI = [1.002, 1.067], P = .048
	Interpretation: Risk of shock estimated to increase 3% for every year of age
**Antibiotics within 24 hrs**	.327
**Immunocompromise/neutropenia**	.950
**WBC**	.430
**Time to effective antibiotics**	.341
**Final combination Abx**	.712
**MDR *P*. *aeruginosa***	.333
**Cancer**	.104
**Burns**	.854
**SOT**	.569
**Non-motile (refers to swimming motility)**	OR = 6.8, 95% CI = [1.37, 51.5], P = .030
**Twitching motility**	.546
**Proteozone**	.811
***exoS* present**	.521
***exoU* present**	.477
**Pyocyanin (continuous)**	.130
**Pyocyanin >10 ug/ml**	.275
**Pyocyanin >18 ug/ml**	OR = 16.9, 95% CI = [2.27, 360], P = .017

## Discussion

The purpose of this study was to examine the role of virulence factors of *P*. *aeruginosa* in outcomes of *Pab*. Our data support a role for high pyocyanin production and non-motility of infecting strains in affecting or predicting outcomes in *Pab*.

The roles of the many virulence factors of *P*. *aeruginosa* have been examined in studies of different mouse models of infection with the general conclusion that *P*. *aeruginosa*‘s virulence is multifactorial and often dependent on the primary site of infection [10–12]. Human studies to confirm any of these roles, particularly in bacteremia are few. However, during the last 2 decades the T3SS has been implicated in poor outcomes in both *Pab* and pneumonia in humans [16, 18, 19, 34]. The presence of the T3 secretion phenotype was correlated with poor outcomes in patients with lung infections or bacteremia. A study that included only bacteremic patients, examined the expression of *exoU* and *exoS*, and implicated both major T3 secreted toxins in death [18]. More recently a larger multicenter study of *Pab* performed an analysis of the role of strains carrying *exoU* and concluded that ExoU may be responsible for deaths occurring in 24–72 h but not for total 30 day mortality [19]. Thus, there appears to be mounting evidence in humans that the ExoU protein plays a role in some deaths due to *Pab*. Our data do not contradict these findings but add to the existing literature. Again, sample size in our study may have precluded the identification of the role of these genes in early deaths.

Pyocyanin, a phenazine pigment produced by *P*. *aeruginosa*, has long been suspected to play a role in the pathogenesis of *P*. *aeruginosa* infections but supportive evidence has been lacking in humans. Our findings that high pyocyanin production *in vitro*, may be predictive of poor outcomes is consistent with work done on this “toxin”. Pyocyanin demonstrates toxic properties towards cells mediated through oxidative stress and the generation of reactive oxygen species [35]. One very important relevant effect is the ability of pyocyanin to cause accelerated neutrophil apoptosis in the lungs resulting in impaired bacterial clearance and significantly lower concentrations of potent neutrophil chemokines [36]. Next, there are animal data that demonstrate the toxic potential of this molecule or related phenazines to the lung as well as their lethal potential in mice [37]. Pyocyanin producing strains produced extensive lobar pneumonia whereas, their isogenic mutants deleted of a gene in the biosynthetic pathway caused mild lung disease. Thus, this molecule in addition to being toxic to the host serves to evade neutrophil. The issue may be raised that these poor early outcomes may not be due to pyocyanin but rather the expression of *exoU*. However, none of the high pyocyanin producers carried the *exoU* gene. In fact, it appears that ExoU producing isolates of *P*. *aerugi*nosa actually produce low amounts of pyocyanin [13].

The most unexpected result from this study was the association of poor outcomes with non-motile *P*. *aeruginosa* isolate*s* especially since motility is essential for virulence in the burn wound model of *P*. *aeruginosa* infection [24, 33]. This seemingly paradoxical observation is however supported by research on the role of motility in phagocytosis and killing of *P*. *aeruginosa*. The polymorphonuclear leucocyte is the first line defense against *P*. *aerug*inosa [38] through non-opsonic phagocytosis which is dependent of the presence of a flagellum [39]. However studies, recently reported, suggest that it is not the flagellum but flagellar motility that is required [40], since the loss of motility, independent of the presence of the flagellum confers *P*. *aeruginosa* with about 100 fold resistance to phagocytosis by neutrophils, macrophages and dendritic cells [40]. Adding to the phagocytic defect, it has now been shown that the absence of flagellar motility results in another neutrophil defect, the inability to release neutrophil extracellular traps (NETs) an alternative mechanism to phagocytosis that is used for killing microbes extracellularly via a DNA scaffold associated with neutrophil granule components [41]. However, we hypothesize that even if there is neutrophil dysfunction, some other virulence factor (s) e.g. ExoU mediates actual toxicity to the host, leading to poor outcomes, since the non-motile isolates produced low amounts of pyocyanin.

Thus, we have defined two other microbial factors that are predictive of poor outcomes in *Pab*, high pyocyanin production and the absence of motility. However, our study has several shortcomings. The small sample size may not have allowed to us identify certain other risk factors for poor outcomes that have been identified in previous studies, such as adequacy of antibiotic therapy, neutropenia/immunosuppression. In addition, we did not examine the phenotypic expression of the T3SS to ascertain whether there were differences in gene expression between strains from SS and non-SS patients. Another caveat should also be stated, our strain evaluation was done *in vitro* thus whether pyocyanin is produced in high amounts *in-vivo* is unknown. Despite these shortcomings, the identification of these two factors in such a small sample size suggests that they play extremely important roles or serve as markers for strains that result in poor outcomes. The common effect of these 2 phenotypes can be summed up as mechanisms to evade phagocytic killing by *P*. *aeruginosa*, the first line of defense in a host. Larger studies to confirm these findings, especially the role of pyocyanin, are warranted, as progress is being made in the development of anti-pyocyanin compounds [42]. It should however be pointed out that the events that occur in blood during Pseudomonas bacteremia are complex and we may just be observing markers for poor outcomes, as exposure of *P*. *aeruginosa* to blood results in the induction and repression of a large number of putative virulence pathways [43].

## Supporting information

S1 Fig Photo of PCR gene products for *exoS*, *toxA*, *lasB* and *exoU* genes electrophoresed through agarose gel containing ethidium bromide and visualized using UV light. This is a representative DNA agarose gel run after PCR analysis of isolate 26–31. Multiple PCRs were run in order to evaluate all 75 isolates. PAK-wild type was the positive control strain for *exoS*, *toxA*, *lasB*, and *plcH* genes. PA-14 laboratory strain was the positive control for *exoU*, *toxA*, *lasB*, and *plcH* genes. The PCRs for *exoS*, *toxA*, *lasB* were run in the same reaction tube using primers for those genes, shown in lane 2–9. PCR reaction using *plcH* primers in lane 10–16. Lanes 1, 17, DNA ladder; lane 2, PAK-wild type strain; lane 3, PA-14 laboratory strain; lane 4–9, isolate 26–31; lane 10, PAK-wild type; lane 11, PA-14; lane 12–16, isolate 26–30. Gene size for each gene encoding a toxin: *exoS* (1361 bp); *exoU* (2063bp); *toxA* (1916bp); *lasB* (1496bp); *plcH* (2192bp).
(TIFF)

S2 Fig Photo of PCR gene products for *plcH* and *exoU* genes electrophoresed through agarose gel containing ethidium bromide and visualized using UV light. This is a representative of the DNA agarose gel run after PCR analysis of isolate 26–31. PAK-wild type and PA-14 were the positive controls for *plcH* gene (see S1 Fig). PA-14 laboratory strain was the positive control for *exoU* gene. Lane 1 and 11, DNA ladder; lane 2, isolate 31 with a band for *plcH* gene; lane 3–10 PCR products of amplification of *exoU* gene; lane 3, Pak-wild type (no band corresponding to *exoU*); lane 4, PA-14; lane 5–10, isolate 26–31. Gene size for each gene encoding a toxin: *exoS* (1361 bp); *exoU* (2063bp); *toxA* (1916bp); *lasB* (1496bp); *plcH* (2192bp).
(TIFF)

S3 Fig Western blot analysis of flagellin production by 8 non-motile clinical isolates. Lane1, isolate 79; lane 2 isolate 77; lane 3, isolate 62; lane 4, isolate 40; lane 5, isolate 35; lane 6, isolate 26; lane 7, isolate 16; lane 8, isolate 9; lane 9, PAK Δflic mutant; lane 10, PAK-*fli*D mutant overexpressing flagellin; lane 11, Pharmacia low-molecular-mass (kilodaltons) markers. Flagellin stains demonstrate different molecular weights due to the fact that there are 2 major flagellin types A and B that have different molecular weights and different degrees of glycosylation. The antibody used detects both types of flagellins.
(TIFF)

S1 Table Primers used for detection of genes encoding the major *Pseudomonas* toxins: *exoS*, *toxA*, *lasB*, *plcH*, and *exoU*. Primers were designed based on the published gene sequences from *Pseudomonas* Genome Database and synthesized by Geno-Mechanix, Gainesville, Florida.
(TIFF)

S1 File (XLS)

## References

[1] Vidal F, Mensa J, Almela M, Martinez JA, Marco F, Casals C, et al. Epidemiology and outcome of Pseudomonas aeruginosa bacteremia, with special emphasis on the influence of antibiotic treatment. Analysis of 189 episodes. Archives of internal medicine. 1996;156(18):2121–6. Epub 1996/10/14. . 8862105

[2] Lodise TP Jr., Patel N, Kwa A, Graves J, Furuno JP, Graffunder E, et al. Predictors of 30-day mortality among patients with Pseudomonas aeruginosa bloodstream infections: impact of delayed appropriate antibiotic selection. Antimicrobial agents and chemotherapy. 2007;51(10):3510–5. Epub 2007/07/25. doi: 10.1128/AAC.00338-07 ; PubMed Central PMCID: PMC2043259. 17646415 PMC2043259

[3] Kang CI, Kim SH, Kim HB, Park SW, Choe YJ, Oh MD, et al. Pseudomonas aeruginosa bacteremia: risk factors for mortality and influence of delayed receipt of effective antimicrobial therapy on clinical outcome. Clinical infectious diseases: an official publication of the Infectious Diseases Society of America. 2003;37(6):745–51. Epub 2003/09/05. doi: 10.1086/377200 . 12955633

[4] Kang CI, Kim SH, Park WB, Lee KD, Kim HB, Kim EC, et al. Bloodstream infections caused by antibiotic-resistant gram-negative bacilli: risk factors for mortality and impact of inappropriate initial antimicrobial therapy on outcome. Antimicrobial agents and chemotherapy. 2005;49(2):760–6. Epub 2005/01/28. doi: 10.1128/AAC.49.2.760-766.2005 ; PubMed Central PMCID: PMC547233. 15673761 PMC547233

[5] Micek ST, Lloyd AE, Ritchie DJ, Reichley RM, Fraser VJ, Kollef MH. Pseudomonas aeruginosa bloodstream infection: importance of appropriate initial antimicrobial treatment. Antimicrobial agents and chemotherapy. 2005;49(4):1306–11. Epub 2005/03/29. doi: 10.1128/AAC.49.4.1306-1311.2005 ; PubMed Central PMCID: PMC1068618. 15793102 PMC1068618

[6] Osmon S, Ward S, Fraser VJ, Kollef MH. Hospital mortality for patients with bacteremia due to Staphylococcus aureus or Pseudomonas aeruginosa. Chest. 2004;125(2):607–16. Epub 2004/02/11. doi: 10.1378/chest.125.2.607 . 14769745

[7] Bodey GP, Jadeja L, Elting L. Pseudomonas bacteremia. Retrospective analysis of 410 episodes. Archives of internal medicine. 1985;145(9):1621–9. Epub 1985/09/01. doi: 10.1001/archinte.145.9.1621 . 3927867

[8] Maschmeyer G, Braveny I. Review of the incidence and prognosis of Pseudomonas aeruginosa infections in cancer patients in the 1990s. European journal of clinical microbiology & infectious diseases: official publication of the European Society of Clinical Microbiology. 2000;19(12):915–25. Epub 2001/02/24. doi: 10.1007/s100960000410 . 11205628

[9] Woods DE, Lam JS, Paranchych W, Speert DP, Campbell M, Godfrey AJ. Correlation of Pseudomonas aeruginosa virulence factors from clinical and environmental isolates with pathogenicity in the neutropenic mouse. Canadian journal of microbiology. 1997;43(6):541–51. Epub 1997/06/01. doi: 10.1139/m97-077 . 9226874

[10] Nicas TI, Iglewski BH. The contribution of exoproducts to virulence of Pseudomonas aeruginosa. Canadian journal of microbiology. 1985;31(4):387–92. Epub 1985/04/01. doi: 10.1139/m85-074 . 2988728

[11] Vasil ML. Pseudomonas aeruginosa: biology, mechanisms of virulence, epidemiology. The Journal of pediatrics. 1986;108(5 Pt 2):800–5. Epub 1986/05/01. doi: 10.1016/s0022-3476(86)80748-x . 3009772

[12] Kurahashi K, Kajikawa O, Sawa T, Ohara M, Gropper MA, Frank DW, et al. Pathogenesis of septic shock in Pseudomonas aeruginosa pneumonia. The Journal of clinical investigation. 1999;104(6):743–50. Epub 1999/09/24. doi: 10.1172/JCI7124 ; PubMed Central PMCID: PMC408437. 10491409 PMC408437

[13] Le Berre R, Nguyen S, Nowak E, Kipnis E, Pierre M, Quenee L, et al. Relative contribution of three main virulence factors in Pseudomonas aeruginosa pneumonia. Critical care medicine. 2011;39(9):2113–20. Epub 2011/05/17. doi: 10.1097/CCM.0b013e31821e899f . 21572326

[14] Jyot J, Balloy V, Jouvion G, Verma A, Touqui L, Huerre M, et al. Type II secretion system of Pseudomonas aeruginosa: in vivo evidence of a significant role in death due to lung infection. The Journal of infectious diseases. 2011;203(10):1369–77. Epub 2011/04/20. doi: 10.1093/infdis/jir045 ; PubMed Central PMCID: PMC3080898. 21502078 PMC3080898

[15] Howell HA, Logan LK, Hauser AR. Type III secretion of ExoU is critical during early Pseudomonas aeruginosa pneumonia. mBio. 2013;4(2):e00032–13. Epub 2013/03/14. doi: 10.1128/mBio.00032-13 ; PubMed Central PMCID: PMC3604777. 23481600 PMC3604777

[16] Roy-Burman A, Savel RH, Racine S, Swanson BL, Revadigar NS, Fujimoto J, et al. Type III protein secretion is associated with death in lower respiratory and systemic Pseudomonas aeruginosa infections. The Journal of infectious diseases. 2001;183(12):1767–74. Epub 2001/05/24. doi: 10.1086/320737 . 11372029

[17] Hauser AR, Cobb E, Bodi M, Mariscal D, Valles J, Engel JN, et al. Type III protein secretion is associated with poor clinical outcomes in patients with ventilator-associated pneumonia caused by Pseudomonas aeruginosa. Critical care medicine. 2002;30(3):521–8. Epub 2002/05/07. doi: 10.1097/00003246-200203000-00005 . 11990909

[18] El-Solh AA, Hattemer A, Hauser AR, Alhajhusain A, Vora H. Clinical outcomes of type III Pseudomonas aeruginosa bacteremia. Critical care medicine. 2012;40(4):1157–63. Epub 2011/11/15. doi: 10.1097/CCM.0b013e3182377906 ; PubMed Central PMCID: PMC3288436. 22080633 PMC3288436

[19] Pena C, Cabot G, Gomez-Zorrilla S, Zamorano L, Ocampo-Sosa A, Murillas J, et al. Influence of virulence genotype and resistance profile in the mortality of Pseudomonas aeruginosa bloodstream infections. Clinical infectious diseases: an official publication of the Infectious Diseases Society of America. 2015;60(4):539–48. Epub 2014/11/08. doi: 10.1093/cid/ciu866 . 25378459

[20] Recio R, Mancheno M, Viedma E, Villa J, Orellana MA, Lora-Tamayo J, et al. Predictors of Mortality in Bloodstream Infections Caused by Pseudomonas aeruginosa and Impact of Antimicrobial Resistance and Bacterial Virulence. Antimicrobial agents and chemotherapy. 2020;64(2). Epub 2019/11/27. doi: 10.1128/AAC.01759-19 ; PubMed Central PMCID: PMC6985728. 31767719 PMC6985728

[21] Recio R, Sanchez-Diener I, Viedma E, Melendez-Carmona MA, Villa J, Orellana MA, et al. Pathogenic characteristics of Pseudomonas aeruginosa bacteraemia isolates in a high-endemicity setting for ST175 and ST235 high-risk clones. European journal of clinical microbiology & infectious diseases: official publication of the European Society of Clinical Microbiology. 2020;39(4):671–8. Epub 2019/12/12. doi: 10.1007/s10096-019-03780-z . 31823150

[22] Bowers DR, Liew YX, Lye DC, Kwa AL, Hsu LY, Tam VH. Outcomes of appropriate empiric combination versus monotherapy for Pseudomonas aeruginosa bacteremia. Antimicrobial agents and chemotherapy. 2013;57(3):1270–4. Epub 2012/12/25. doi: 10.1128/AAC.02235-12 ; PubMed Central PMCID: PMC3591924. 23263001 PMC3591924

[23] Feltman H, Schulert G, Khan S, Jain M, Peterson L, Hauser AR. Prevalence of type III secretion genes in clinical and environmental isolates of Pseudomonas aeruginosa. Microbiology. 2001;147(Pt 10):2659–69. Epub 2001/09/29. doi: 10.1099/00221287-147-10-2659 . 11577145

[24] Arora SK, Neely AN, Blair B, Lory S, Ramphal R. Role of motility and flagellin glycosylation in the pathogenesis of Pseudomonas aeruginosa burn wound infections. Infection and immunity. 2005;73(7):4395–8. Epub 2005/06/24. doi: 10.1128/IAI.73.7.4395-4398.2005 ; PubMed Central PMCID: PMC1168557. 15972536 PMC1168557

[25] Totten PA, Lory S. Characterization of the type a flagellin gene from Pseudomonas aeruginosa PAK. Journal of bacteriology. 1990;172(12):7188–99. Epub 1990/12/01. doi: 10.1128/jb.172.12.7188-7199.1990 ; PubMed Central PMCID: PMC210844. 2123866 PMC210844

[26] Rashid MH, Kornberg A. Inorganic polyphosphate is needed for swimming, swarming, and twitching motilities of Pseudomonas aeruginosa. Proceedings of the National Academy of Sciences of the United States of America. 2000;97(9):4885–90. Epub 2000/04/12. doi: 10.1073/pnas.060030097 ; PubMed Central PMCID: PMC18327. 10758151 PMC18327

[27] Sokol PA, Ohman DE, Iglewski BH. A more sensitive plate assay for detection of protease production by Pseudomanas aeruginosa. Journal of clinical microbiology. 1979;9(4):538–40. Epub 1979/04/01. doi: 10.1128/jcm.9.4.538-540.1979 ; PubMed Central PMCID: PMC273070. 110831 PMC273070

[28] Essar DW, Eberly L, Hadero A, Crawford IP. Identification and characterization of genes for a second anthranilate synthase in Pseudomonas aeruginosa: interchangeability of the two anthranilate synthases and evolutionary implications. Journal of bacteriology. 1990;172(2):884–900. Epub 1990/02/01. doi: 10.1128/jb.172.2.884-900.1990 ; PubMed Central PMCID: PMC208517. 2153661 PMC208517

[29] Balloy V, Verma A, Kuravi S, Si-Tahar M, Chignard M, Ramphal R. The role of flagellin versus motility in acute lung disease caused by Pseudomonas aeruginosa. The Journal of infectious diseases. 2007;196(2):289–96. Epub 2007/06/16. doi: 10.1086/518610 . 17570117

[30] Dasgupta N, Wolfgang MC, Goodman AL, Arora SK, Jyot J, Lory S, et al. A four-tiered transcriptional regulatory circuit controls flagellar biogenesis in Pseudomonas aeruginosa. Molecular microbiology. 2003;50(3):809–24. Epub 2003/11/18. doi: 10.1046/j.1365-2958.2003.03740.x . 14617143

[31] Dasgupta N, Arora SK, Ramphal R. fleN, a gene that regulates flagellar number in Pseudomonas aeruginosa. Journal of bacteriology. 2000;182(2):357–64. Epub 2000/01/12. doi: 10.1128/JB.182.2.357-364.2000 ; PubMed Central PMCID: PMC94283. 10629180 PMC94283

[32] Sonawane A, Jyot J, Ramphal R. Pseudomonas aeruginosa LecB is involved in pilus biogenesis and protease IV activity but not in adhesion to respiratory mucins. Infection and immunity. 2006;74(12):7035–9. Epub 2006/10/04. doi: 10.1128/IAI.00551-06 ; PubMed Central PMCID: PMC1698087. 17015462 PMC1698087

[33] Montie TC, Doyle-Huntzinger D, Craven RC, Holder IA. Loss of virulence associated with absence of flagellum in an isogenic mutant of Pseudomonas aeruginosa in the burned-mouse model. Infection and immunity. 1982;38(3):1296–8. Epub 1982/12/01. doi: 10.1128/iai.38.3.1296-1298.1982 ; PubMed Central PMCID: PMC347889. 6818148 PMC347889

[34] Shaver CM, Hauser AR. Relative contributions of Pseudomonas aeruginosa ExoU, ExoS, and ExoT to virulence in the lung. Infection and immunity. 2004;72(12):6969–77. Epub 2004/11/24. doi: 10.1128/IAI.72.12.6969-6977.2004 ; PubMed Central PMCID: PMC529154. 15557619 PMC529154

[35] Hall S, McDermott C, Anoopkumar-Dukie S, McFarland AJ, Forbes A, Perkins AV, et al. Cellular Effects of Pyocyanin, a Secreted Virulence Factor of Pseudomonas aeruginosa. Toxins. 2016;8(8). Epub 2016/08/16. doi: 10.3390/toxins8080236 ; PubMed Central PMCID: PMC4999852. 27517959 PMC4999852

[36] Allen L, Dockrell DH, Pattery T, Lee DG, Cornelis P, Hellewell PG, et al. Pyocyanin production by Pseudomonas aeruginosa induces neutrophil apoptosis and impairs neutrophil-mediated host defenses in vivo. J Immunol. 2005;174(6):3643–9. Epub 2005/03/08. doi: 10.4049/jimmunol.174.6.3643 . 15749902

[37] Lau GW, Ran H, Kong F, Hassett DJ, Mavrodi D. Pseudomonas aeruginosa pyocyanin is critical for lung infection in mice. Infection and immunity. 2004;72(7):4275–8. Epub 2004/06/24. doi: 10.1128/IAI.72.7.4275-4278.2004 ; PubMed Central PMCID: PMC427412. 15213173 PMC427412

[38] Koh AY, Priebe GP, Ray C, Van Rooijen N, Pier GB. Inescapable need for neutrophils as mediators of cellular innate immunity to acute Pseudomonas aeruginosa pneumonia. Infection and immunity. 2009;77(12):5300–10. Epub 2009/10/07. doi: 10.1128/IAI.00501-09 ; PubMed Central PMCID: PMC2786465. 19805527 PMC2786465

[39] Mahenthiralingam E, Speert DP. Nonopsonic phagocytosis of Pseudomonas aeruginosa by macrophages and polymorphonuclear leukocytes requires the presence of the bacterial flagellum. Infection and immunity. 1995;63(11):4519–23. Epub 1995/11/01. doi: 10.1128/iai.63.11.4519-4523.1995 ; PubMed Central PMCID: PMC173644. 7591095 PMC173644

[40] Amiel E, Lovewell RR, O’Toole GA, Hogan DA, Berwin B. Pseudomonas aeruginosa evasion of phagocytosis is mediated by loss of swimming motility and is independent of flagellum expression. Infection and immunity. 2010;78(7):2937–45. Epub 2010/05/12. doi: 10.1128/IAI.00144-10 ; PubMed Central PMCID: PMC2897393. 20457788 PMC2897393

[41] Floyd M, Winn M, Cullen C, Sil P, Chassaing B, Yoo DG, et al. Swimming Motility Mediates the Formation of Neutrophil Extracellular Traps Induced by Flagellated Pseudomonas aeruginosa. PLoS pathogens. 2016;12(11):e1005987. Epub 2016/11/18. doi: 10.1371/journal.ppat.1005987 ; PubMed Central PMCID: PMC5113990. 27855208 PMC5113990

[42] Miller LC, O’Loughlin CT, Zhang Z, Siryaporn A, Silpe JE, Bassler BL, et al. Development of potent inhibitors of pyocyanin production in Pseudomonas aeruginosa. Journal of medicinal chemistry. 2015;58(3):1298–306. Epub 2015/01/20. doi: 10.1021/jm5015082 ; PubMed Central PMCID: PMC4332565. 25597392 PMC4332565

[43] Beasley KL, Cristy SA, Elmassry MM, Dzvova N, Colmer-Hamood JA, Hamood AN. During bacteremia, Pseudomonas aeruginosa PAO1 adapts by altering the expression of numerous virulence genes including those involved in quorum sensing. PLoS One. 2020;15(10):e0240351. Epub 2020/10/16. doi: 10.1371/journal.pone.0240351 ; PubMed Central PMCID: PMC7561203. 33057423 PMC7561203

