# Supplementary figures and images for: High pyocyanin production and non-motility of Pseudomonas aeruginosa isolates are correlated with septic shock or death in bacteremic patients
Source: PLoS One. 2021 Jun 11;16(6):e0253259. doi: 10.1371/journal.pone.0253259 (PMC8195364; doi:10.1371/journal.pone.0253259)

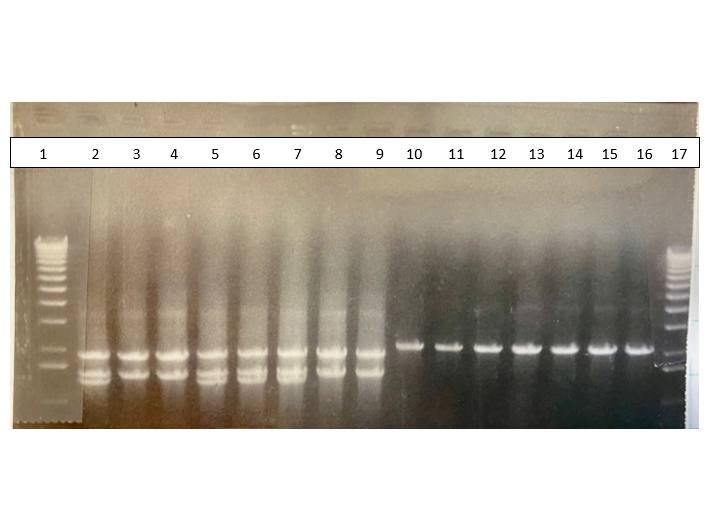

Supplement: S1 Fig — This is a representative DNA agarose gel run after PCR analysis of isolate 26–31. Multiple PCRs were run in order to evaluate all 75 isolates. PAK-wild type was the positive control strain for exoS, toxA, lasB, and plcH genes. PA-14 laboratory strain was the positive control for exoU, toxA, lasB, and plcH genes. The PCRs for exoS, toxA, lasB were run in the same reaction tube using primers for those genes, shown in lane 2–9. PCR reaction using plcH primers in lane 10–16. Lanes 1, 17, DNA ladder; lane 2, PAK-wild type strain; lane 3, PA-14 laboratory strain; lane 4–9, isolate 26–31; lane 10, PAK-wild type; lane 11, PA-14; lane 12–16, isolate 26–30. Gene size for each gene encoding a toxin: exoS (1361 bp); exoU (2063bp); toxA (1916bp); lasB (1496bp); plcH (2192bp). (TIFF) [file pone.0253259.s001.tiff]

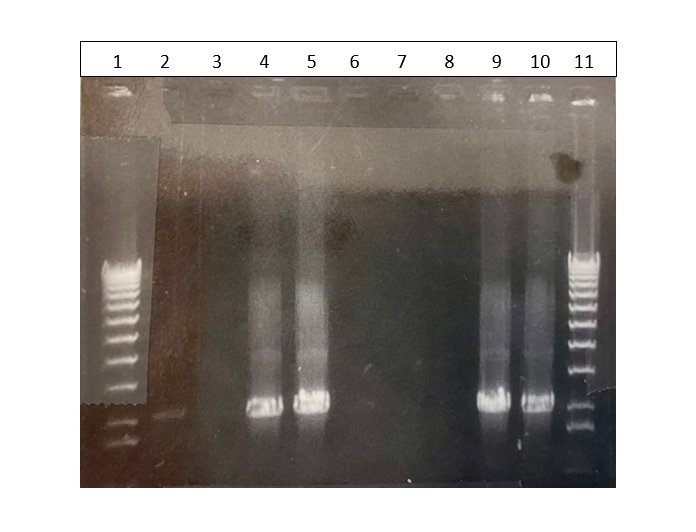

Supplement: S2 Fig — This is a representative of the DNA agarose gel run after PCR analysis of isolate 26–31. PAK-wild type and PA-14 were the positive controls for plcH gene (see S1 Fig). PA-14 laboratory strain was the positive control for exoU gene. Lane 1 and 11, DNA ladder; lane 2, isolate 31 with a band for plcH gene; lane 3–10 PCR products of amplification of exoU gene; lane 3, Pak-wild type (no band corresponding to exoU); lane 4, PA-14; lane 5–10, isolate 26–31. Gene size for each gene encoding a toxin: exoS (1361 bp); exoU (2063bp); toxA (1916bp); lasB (1496bp); plcH (2192bp). (TIFF) [file pone.0253259.s002.tiff]

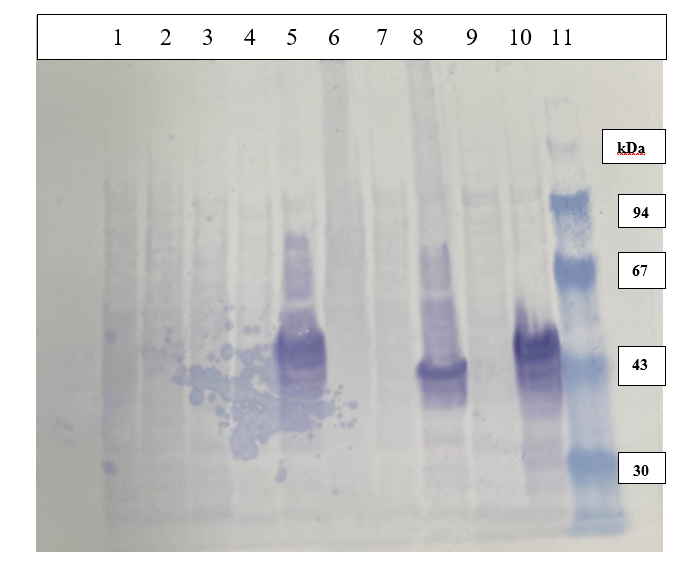

Supplement: S3 Fig — Lane1, isolate 79; lane 2 isolate 77; lane 3, isolate 62; lane 4, isolate 40; lane 5, isolate 35; lane 6, isolate 26; lane 7, isolate 16; lane 8, isolate 9; lane 9, PAK Δflic mutant; lane 10, PAK-fliD mutant overexpressing flagellin; lane 11, Pharmacia low-molecular-mass (kilodaltons) markers. Flagellin stains demonstrate different molecular weights due to the fact that there are 2 major flagellin types A and B that have different molecular weights and different degrees of glycosylation. The antibody used detects both types of flagellins. (TIFF) [file pone.0253259.s003.tiff]

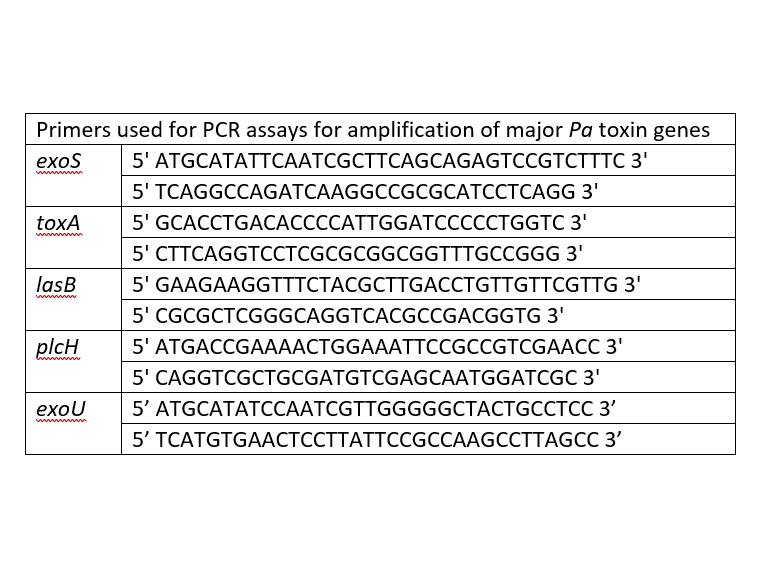

Supplement: S1 Table — Primers were designed based on the published gene sequences from Pseudomonas Genome Database and synthesized by Geno-Mechanix, Gainesville, Florida. (TIFF) [file pone.0253259.s004.tiff]
